# Association of cerebrovascular dysfunction with the development of Alzheimer’s disease-like pathology in OXYS rats

**DOI:** 10.1186/s12864-018-4480-9

**Published:** 2018-02-09

**Authors:** Natalia A. Stefanova, Kseniya Yi Maksimova, Ekaterina A. Rudnitskaya, Natalia A. Muraleva, Nataliya G. Kolosova

**Affiliations:** 1 Institute of Cytology and Genetics, Novosibirsk, Russia; 2 Siberian State Medical University, Tomsk, Russia; 3 Novosibirsk State University, Novosibirsk, Russia

**Keywords:** Alzheimer’s disease, Cerebrovascular dysfunction, RNA sequencing, OXYS rats

## Abstract

**Background:**

Cerebrovascular dysfunction plays a critical role in the pathogenesis of Alzheimer’s disease (AD): the most common cause of dementia in the elderly. The involvement of neurovasculature disorders in the progression of AD is now increasingly appreciated, but whether they represent initial factors or late-stage pathological changes during the disease is unclear. Using senescence-accelerated OXYS rats, which simulate key characteristics of sporadic AD, we evaluated contributions of cerebrovascular alterations to the disease development. At preclinical, early, and advanced stages of AD-like pathology, in the hippocampus of OXYS and Wistar (control) rats, we evaluated (i) the blood vessel state by histological and electron-microscopic analyses; (ii) differences in gene expression according to RNA sequencing (RNA-Seq) to identify the metabolic processes and pathways associated with blood vessel function; (iii) the amount of vascular endothelial growth factor (VEGF) by western blot and immunohistochemical analysis.

**Results:**

We observed a loss of hippocampal blood vessel density and ultrastructural changes of those blood vessels in OXYS rats at the early stage of AD-like pathology. There were significant alterations in the vessels and downregulation of VEGF with an increased amount of amyloid β_1–42_ there at the advanced stage of the disease. According to RNA-Seq data analysis, major alterations in cerebrovascular processes of OXYS rats were associated with blood vessel development, circulatory system processes, the VEGF signaling pathway, and vascular smooth muscle contraction. At preclinical and early stages of the AD-like pathology, these processes were upregulated and then downregulated with age. At the advanced stage in OXYS rats, differentially expressed genes (DEGs) were associated with downregulation of cerebrovascular function as compared to Wistar rats. Among the 46 DEGs at the preclinical stage of the disease, 28 DEGs at the early stage, and among 85 DEGs at the advanced stage, using functional analysis and gene network construction, we identified genes (*Nos1*, *P2rx4*, *Pla2g6*, and *Bdkrb2*) probably playing a significant role in the development of cerebrovascular dysfunction in OXYS rats.

**Conclusions:**

Changes in expression of the genes functionally associated with cerebrovascular processes already in the early period of life may contribute to the development of AD-like pathology in OXYS rats.

**Electronic supplementary material:**

The online version of this article (10.1186/s12864-018-4480-9) contains supplementary material, which is available to authorized users.

## Background

Vascular dysfunction is a universal feature of aging that contributes to the cognitive decline and neurodegeneration observed in Alzheimer’s disease (AD): the most common cause of dementia in the elderly [1, 2]. AD is characterized by a gradual decline in memory and cognition that correlate with synaptic dysfunction and neuronal loss. It was suggested over 30 years ago that vascular defects present in AD may be important for the development of the disease [3]. The role of a neurovasculature disorder in the progression of AD is now becoming more fully appreciated. It is clear that microvascular deficits diminish cerebral blood flow and, consequently, the brain’s supply of oxygen, energy, substrates, and nutrients. Moreover, such deficits impair the clearance of neurotoxic molecules, such as amyloid β (Aβ), which are released into the brain interstitial fluid and accumulate in the parenchyma and vessel walls. Patients with AD frequently show focal changes in brain microcirculation. These changes include alterations in density and morphology of cerebral microvasculature, increased endothelial pinocytosis, a decrease in mitochondrial content, accumulation of collagen and perlecans in the basement membrane, loss of tight junctions and/or adherens junctions, and a blood-brain barrier breakdown with leakage of blood-borne molecules [4, 5].

Multiple epidemiological studies have shown a remarkable overlap among risk factors for a cerebrovascular disorder and sporadic, late-onset AD [6], but whether they represent initial factors or late-stage pathological changes during the disease is still a matter of debate. Growing data from brain imaging studies in humans and animal models suggest that cerebrovascular dysfunction may precede a cognitive decline and the onset of neurodegenerative changes in AD and animal models, yet the molecular and cellular mechanisms and targets that underlie the contribution of vascular disease to AD are not well understood [5].

Because in humans, the research on the pathogenesis of AD, especially early asymptomatic stages, is problematic, there is a need for animal models that closely mirror the human pathology. There is evidence that a suitable model of sporadic form of AD is senescence-accelerated OXYS rats. These animals spontaneously develop all key signs of AD such as structural neurodegenerative alterations, neuronal loss, synaptic damage, disturbances of the neurotrophic supply, Aβ_1–42_ peptide accumulation and tau hyperphosphorylation in the hippocampus, and impairment of the learning ability and memory [7–10].

Using MRI, we showed that at the age of 12 months, when AD was progressing, OXYS rats had structural and functional alterations in the cerebral blood flow typical of chronic ischemia such as reduced cerebral blood flow and a decline of cerebrovascular reactivity in response to a vasodilatory challenge, also defined as the cerebrovascular reserve [11, 12], as in patients with AD. In addition, we found that OXYS rats show altered hemorheological properties of blood resulting from abnormal red blood cell deformability and aggregation that are regarded as one of the mechanisms underlying the complex etiology of AD [13], impairing the oxygen transport efficiency of blood in AD.

In this study, we assessed the mechanisms of cerebrovascular dysfunction and its contributions to the development of AD-like pathology in OXYS rats. We compared these rats with the control Wistar rats at different stages of the disease in OXYS rats, including the preclinical stage. We used histological and electron-microscopic analyses to evaluate cerebrovascular alterations during development of the signs of AD in OXYS rats, a bioinformatic analysis of RNA-Seq data to identify the metabolic processes and pathways involved in the development of cerebrovascular dysfunction, and quantification of vascular endothelial growth factor (VEGF), which is a neuroprotective cytokine promoting neurogenesis and angiogenesis in the brain.

## Results

### Structural cerebrovascular alterations in the hippocampus of OXYS rats

**Fig. 1:**
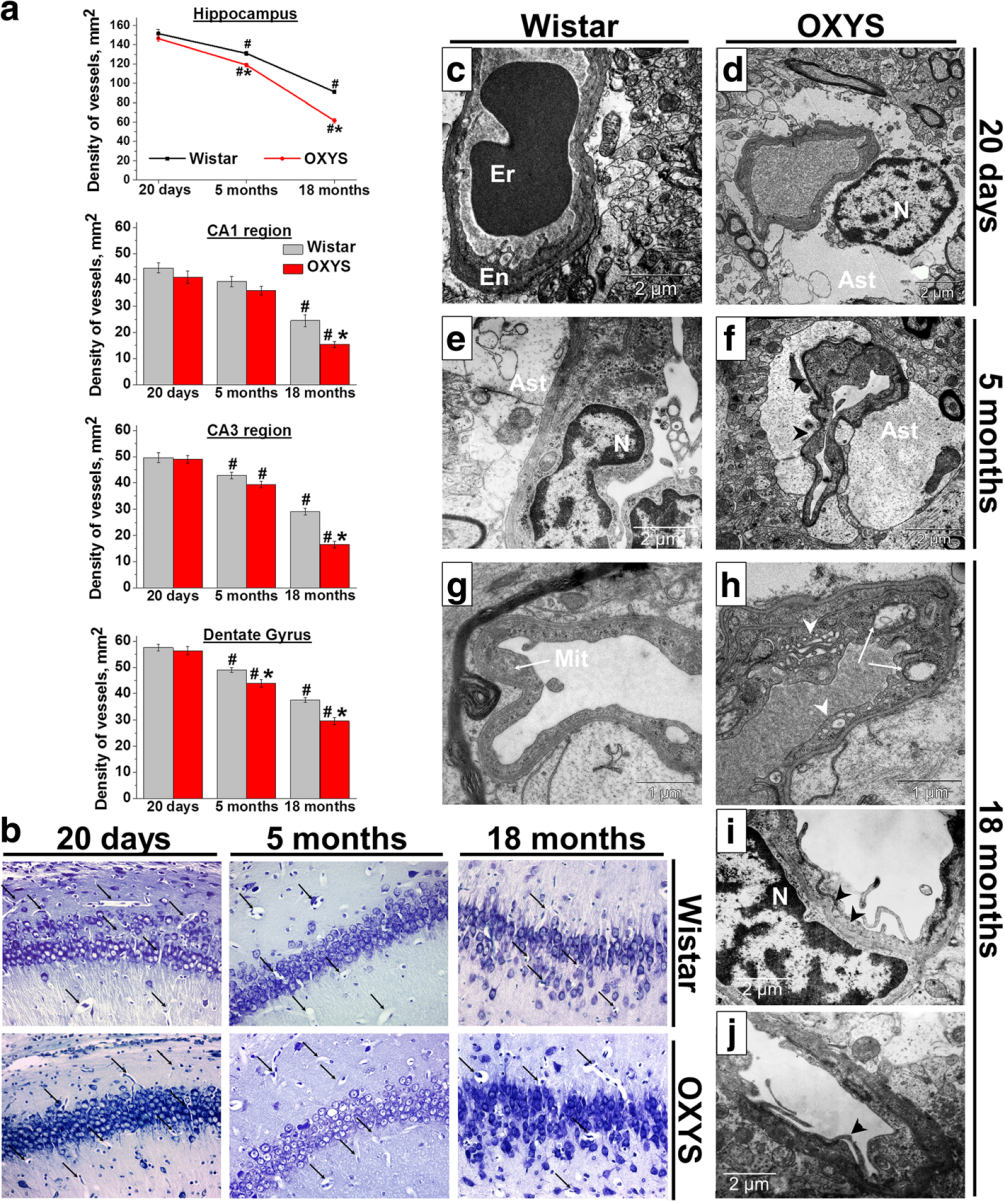
Density of vessels decreased with age in all examined hippocampal regions (CA1 and CA3 regions and dentate gyrus) of Wistar and OXYS rats (**a**) with a more significant age-related decrease in OXYS rats: total blood vessel density in the hippocampus was lower in OXYS rats compared to Wistar rats starting from 5 months of age. ^#^differences with previous age, *p* < 0.05; *differences with age-matched Wistar rats, *p* < 0.05. **b** Density of blood vessels in the CA1 region of the hippocampus of Wistar and OXYS rats decreased with age. Magnification ×20. The arrows indicate blood vessels. The electron micrographs (**c**–**j**) show structural organization of the vessels of 20-day-old and 5- and 18-month-old Wistar and OXYS rats. **c** A morphologically unchanged vessel with an erythrocyte (Er) in the lumen. En: the cytoplasm of an endotheliocyte. **d** A morphologically unchanged vessel. N: the nucleus of an astrocyte (Ast). **e** A morphologically unchanged vessel. N: a nucleus of the endotheliocyte; Ast: the processes of an astrocyte. **f** The arrowheads indicate an increase in electronic density of the basal membrane of the endotheliocyte. Ast: the process of an astrocyte. **g** The destructively altered mitochondria (Mit) in the cytoplasm of an endotheliocyte (indicated by the arrow). **h** Large vesicles in the cytoplasm of an endotheliocyte (indicated by arrows). Massively swollen mitochondria and expansion of the Golgi complex cisterns (indicated by arrowheads). **i** The arrowheads indicate thickening and folding of the basement membrane of an endotheliocyte. N: the nucleus of the pericyte. **j** The arrowhead indicates osmiophilic conglomerates in the cytoplasm of an endotheliocyte

To evaluate cerebrovascular alterations during the development of AD-like pathology in OXYS rats, we conducted comparative analysis of the density and ultrastructural state of blood vessels in the hippocampal СА1 and СА3 regions and dentate gyrus in OXYS and Wistar rats at the age of 20 days and 5 and 18 months. We found that total blood vessel density in the hippocampus was lower in OXYS rats (F_1,54_ = 49.2, *p* < 0.0001). The maximal density of blood vessels in the hippocampus of OXYS and Wistar rats was observed at the age of 20 days, and this density decreased with age in both rat strains (F_2,54_ = 370.7, *p* < 0.0001; Fig. 1a, b). When we analyzed the vessel density in the hippocampal СА1 and СА3 regions and dentate gyrus, we found that in OXYS rats, a significant decrease of this parameter occurred at age 5 months in the dentate gyrus and at the age of 18 months in all hippocampal regions as compared to Wistar rats (*p* < 0.05; Fig. 1a).

According to electron-microscopic analysis (Fig. 1e–j), in contrast to Wistar rats, 5-month-old OXYS rats showed the first changes in structural organization of the vessels, such as an increase in electron density of the basal membrane of endotheliocytes (Fig. 1f) and hyperchromatic nuclei of endotheliocytes. By the age of 18 months, the degenerative changes in hippocampal vessels of OXYS rats had progressed and were characterized by thickening of the wall and a hyperchromatic endothelial nucleus, the cytoplasm of endotheliocytes contained vacuoles, and the membrane formed folds (Fig. 1h, g). Besides, in the cytoplasm of endotheliocytes, large vesicles formed, probably due to destructive changes in the organelles (mitochondria and the endoplasmic reticulum; Fig. 1j). In addition, the pericytes contained lipid aggregates in the cytoplasm, a large number of lysosomes, and low content of organelles. In the mitochondria of pericytes, we often observed destruction of cristae. Thus, these data indicate a hippocampal blood vessel density loss and ultrastructural changes in those blood vessels in OXYS rats in the period of active development of AD-like pathology, and significant alterations of these vessels at the progressive stage of the disease.

### Differential expression of genes in the hippocampus of OXYS rats

To identify the pathways and biological functions involved in the development of cerebrovascular dysfunction in OXYS rats, we used RNA-Seq to obtain the lists of differentially expressed genes (DEGs) in the hippocampus of 20-day-old and 5- and 18-month-old OXYS rats compared to age-matched Wistar rats. Differential expression of genes was evaluated using the DESeq software [14]. We identified 841 protein-coding mRNAs (at *p* < 0.01) that are differentially expressed in 20-day-old OXYS rats compared with age-matched Wistar rats; among those genes, 281 were downregulated, and 560 were upregulated. There were 489 genes that are differentially expressed in 5-month-old OXYS rats compared with age-matched Wistar rats; among these genes, 209 were downregulated, and 280 were upregulated. At 18 months of age, there were 1324 DEGs; among them, 750 were downregulated, and 574 were upregulated in OXYS rats.

### Functional annotation of DEGs associated with cerebrovascular dysfunction in OXYS rats

Differentially expressed protein-coding genes were functionally annotated by means of Gene Ontology (GO) biological process categories and pathways as described in Methods. According to DAVID, at the age of 20 days in OXYS rats, seven GO terms (*p* < 0.05) were found to be associated with blood vessel processes; among these genes, four were upregulated (Additional file 1). In particular, GO terms were enriched for functional categories explicitly related to the blood circulation and blood pressure. At 5 months of age, there were two upregulated GO terms related to the blood vessel development. At 18 months of age, there were nine GO terms; among them, five were downregulated and were enriched for genes belonging to processes involved in blood vessel development and hemopoiesis (Additional file 1). In summary, there are 19 DEGs involved in biological processes associated with hippocampal blood vessels in 20-day-old OXYS rats; among these genes, 3 were downregulated, and 16 were upregulated. At the age of 5 months, there were 8 upregulated DEGs in OXYS rats associated with blood vessel processes. At the age of 18 months, among the 65 DEGs associated with blood vessel processes, mRNA expression of 26 genes was increased in OXYS rats and decreased for the other 39 genes, in comparison with Wistar rats (Additional file 2). Interstrain differences in the expression levels of genes *Cxcl12* and *Itga7* were detected at 5 and 18 months, and for the 7 common genes, interstrain differences were observed between ages 20 days and 18 months (*Agt*, *Bdkrb2*, *Gaa*, *Nos1*, *P2rx4*, *Pcsk5*, and *Ptgs1*), the expression of all genes increased, except for gene *Bdkrb2*.

In KEGG pathway analysis, we found 3 pathways (*p* < 0.05) associated with blood vessel processes in OXYS rats: the vascular smooth muscle contraction pathway was upregulated in OXYS rats at the age of 20 days and 5 months; the VEGF signaling pathway was upregulated at the age of 20 days and downregulated at the age of 18 months; the hematopoietic cell lineage pathway was downregulated at the age of 5 and 18 months (Additional file 2). Note that interstrain differences in the high expression level of gene *Pla2g6*, which is involved in the VEGF signaling pathway and vascular smooth muscle contraction, were observed at all 3 ages.

### Age-associated transcriptome changes that are involved in cerebrovascular function in OXYS and Wistar rats

**Fig. 2:**
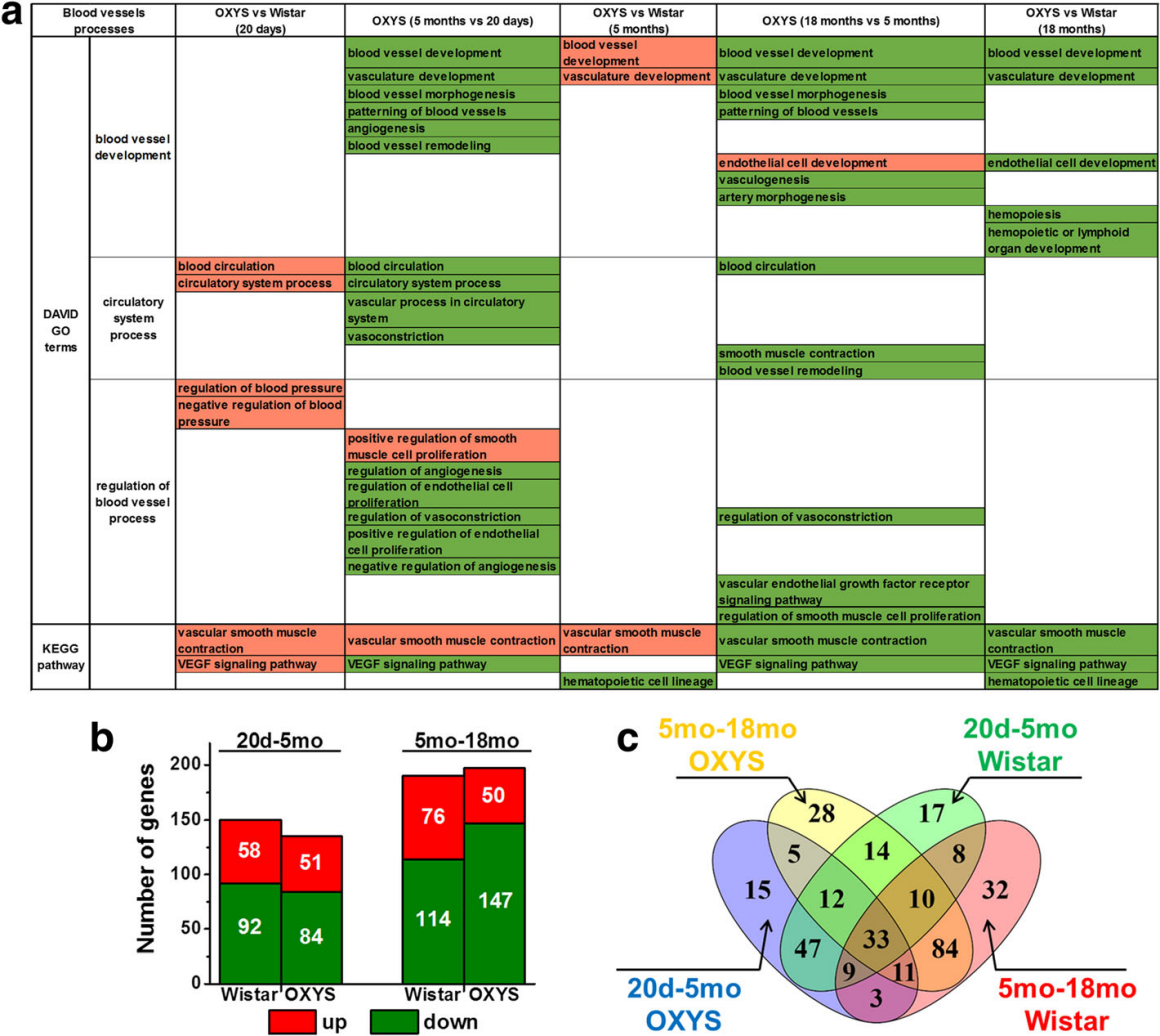
**a** Lists of GO terms associated with blood vessels (according to DAVID) and with pathways (according to KEGG), which undergo changes in the hippocampus of OXYS rats with age, as compared to age-matched Wistar rats. GO terms and pathways are marked red if upregulated and green if downregulated. **b** The number of genes—involved in GO terms (according to DAVID) and in pathways (according to KEGG)—that change expression with age in OXYS and Wistar rats (d: days, mo: months). **c** The Venn diagram shows overlapping sets of genes that change expression with age in OXYS and Wistar rats

We found that in both rat strains, the genes with significantly changed expression with age were associated with a sufficiently large number of GO terms (*p* < 0.05) that are involved in blood vessel processes according to DAVID (Additional file 1). Among these genes, in OXYS and Wistar rats, the genes significantly downregulated between the ages of 20 days and 5 months as well as between the ages of 5 and 18 months were associated with blood vessel development, blood circulation, and its regulation (Fig. 2a). As for the KEGG pathway analysis, we found that in Wistar rats with age, the DEGs were associated with a change in vascular smooth muscle contraction, in the VEGF signaling pathway, and in the hematopoietic cell lineage pathway (Additional file 1). Whereas in OXYS rats, the vascular smooth muscle contraction pathway was significantly downregulated between the ages of 5 and 18 months, and the VEGF signaling pathway was downregulated in both age periods (Fig. 2a; Additional file 1). For a combined analysis, we obtained a total list of DEGs involved in GO terms according to DAVID and in pathways according to KEGG in OXYS and Wistar rats in both age periods (Additional file 1). We found, that in OXYS rats, 135 genes significantly changed expression between the ages of 20 days and 5 months (of these, 84 were downregulated, and 51 were upregulated), and 197 genes changed expression between the ages of 5 and 18 months (of these, 147 were downregulated, and 50 were upregulated; Fig. 2b). In Wistar rats, 150 genes significantly changed expression between the ages of 20 days and 5 months (of these, 92 were downregulated, and 58 were upregulated), and 190 genes changed expression levels between the ages of 5 and 18 months (of these, 114 were downregulated, and 76 were upregulated; Fig. 2b). Figure 2c shows a Venn diagram visualizing the overlaps among the lists of DEGs associated with blood vessel processes in OXYS and Wistar rats in the two periods. In OXYS rats, between the ages of 20 days and 5 months, 15 genes exclusively changing expression were mainly related to blood circulation and regulation of blood pressure (*Npy*, *Stat1*, *Cdc42*, *Tac1*, *Apln*, and *Cyb11b2*; Fig. 2c; Additional file 2). In Wistar rats during the same period, there were 17 genes that exclusively changed expression; they were mainly related to blood vessel morphogenesis and regulation of angiogenesis (*Mylk*, *Prkx*, *Epha2*, *Adrb2*, *Serpine1*, *Notch1*, *Dll4*, and *Vegfc*). In OXYS rats, between the ages of 5 and 18 months, 28 genes exclusively changing expression levels were mainly related to blood circulation and regulation of blood vessel size (*Agt*, *Htr2a*, *Smad5*, *Gclm*, *P2rx4*, *Npy1r*, *Cacna1b*, and *Angpt1*). By contrast, in Wistar rats during the same period, there were 32 genes that exclusively changed expression; they were mainly related to the regulation of lymphocyte proliferation and of immune responses (*Il2ra*, *Il15*, *Cd38*, *Cd74*, and *Serpine1*). Only in OXYS rats, in both age groups, changed expression level were observed for 5 genes, 3 of them are associated with blood vessel morphogenesis (*Cited1*, *Plxnd*, and *Itgav*). In Wistar rats, among 8 DEGs in both age groups, the change of expression of 4 genes was also associated with blood vessel morphogenesis (*Gja5*, *Ctgf*, *Apold1*, and *Itga7*; Fig. 2c; Additional file 2).

### Major altered cerebrovascular processes in OXYS rats and construction of a gene network

**Fig. 3:**
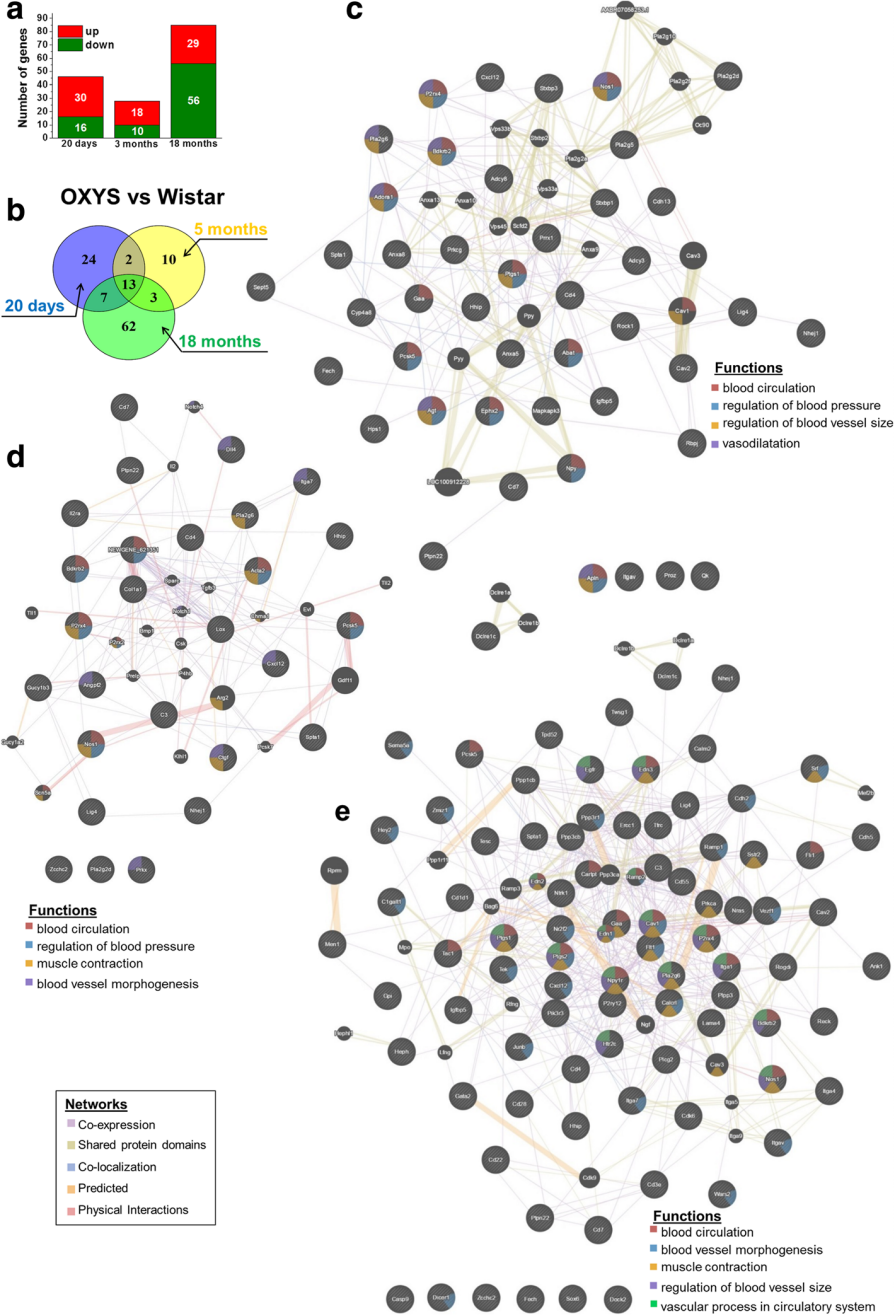
**a** The number of DEGs associated with blood vessel processes in the hippocampus of 20-day-old and 5- and 18-month-old OXYS rats compared to age-matched Wistar rats. **b** The Venn diagram shows overlapping sets of DEGs in OXYS rats compared to age-matched Wistar rats. **c–e** Interaction networks for DEGs in OXYS rats at the age of 20 days (**c**), 5 months (**d**), and 18 months (**e**) as compared to age-matched Wistar rats

Using the DAVID and KEGG analyses, we found that in 20-day-old and 5-month-old OXYS rats, cerebrovascular processes and pathways are upregulated (Fig. 2a). The genes that are differentially expressed during aging were downregulated, and the results revealed that at the age of 18 months in OXYS rats, DEGs were associated with downregulation of cerebrovascular function as compared with Wistar rats. Among these processes, the most significant alterations are associated with blood vessel development, circulatory system processes, and pathways associated with VEGF signaling, vascular smooth muscle contraction, and hematopoietic cell lineages (Fig. 2a). We next compiled a total list of DEGs involved in GO processes and pathways in OXYS rats in 3 age groups (Additional file 1). Already at the age of 20 days in OXYS rats, there are 46 DEGs (16 downregulated and 30 upregulated genes). At the age of 5 months, there were 28 DEGs (10 downregulated and 18 upregulated genes) in OXYS rats associated with blood vessel processes. At the age of 18 months, of the 85 DEGs associated with blood vessel processes, mRNA expression of 56 genes was decreased in OXYS rats and increased for the other 29 genes, in comparison with Wistar rats (Fig. 3a; Additional file 1). Interstrain differences in the expression levels of 13 genes were detected at the ages of 20 days and 5 and 18 months. It should be noted that mRNA levels of *Cxcl12*, *Nhej1*, *Nos1*, *P2rx4*, *Pcsk5*, *Pla2g6*, and *Ptpn22* increased, and the expression of *Cd4*, *Cd7*, *Hhip*, *Lig4*, and *Spta1* decreased in OXYS rats at all 3 ages. Only expression of the *Bdkrb2* gene decreased in 20-day-old and 18-month-old OXYS rats and increased in 5-month-old rats. In addition, interstrain differences in the expression levels of upregulated genes *Itgam* and *Pla2g2d* were observed at 20 days and 5 months, and for the upregulated gene *Itga7* and downregulated genes *C3* and *Zcchc2* interstrain differences in the expression levels were detected at ages 5 and 18 months. For the 7 common genes that were found between ages 20 days and 18 months, the expression of *Fech*, *Gaa*, *Igfbp5*, and *Ptgs1* increased and the expression of *Cav2*, *Dclre1c*, and *Itgav* diminished.

We next used GeneMANIA to construct the interaction networks for the analysis of transcriptomic changes associated with functional categories of cerebrovascular dysfunction during the AD-like pathology development (Fig. 3c–e). It is evident that most of the genes are directly or indirectly related to one another. The GeneMANIA results on enrichment with GO terms for the network’s members are presented in Additional file 3. Among the most enriched GO terms were the regulation of blood pressure (*p* < 4.85E–10), blood circulation (*p* < 1.48E–9), vasodilation (*p* < 4.67E–7), and regulation of blood vessel size (*p* < 1.43E–6) in the gene set of 20-day-old rats (Fig. 3c); blood circulation (*p* < 2.85E–4), the regulation of blood pressure (p < 2.85E–4), muscle contraction (*p* < 1.08E–5), and blood vessel morphogenesis (*p* < 5.98E–4) in the gene set of 5-month-old rats (Fig. 3d); and blood vessel morphogenesis (*p* < 2.22E–17), blood circulation (*p* < 2.16E–11), muscle contraction (*p* < 9.49E–13), regulation of blood vessel size (*p* < 1.24E–11), and a vascular process in circulatory system (*p* < 4.06E–12) in the gene set of 18-month-old OXYS rats (Fig. 3e). Analysis of the gene networks in 20-day-old and 5- and 18-month-old animals revealed that the common genes probably play a significant role in the regulation of blood vessel processes in the hippocampus of OXYS rats during development of AD-like pathology: *Nos1*, *P2rx4*, *Pla2g6*, and *Bdkrb2*. Taken together, these results indicate that in OXYS rats, the development of AD signs is preceded by changes in the expression of hippocampal genes involved in cerebrovascular processes; these changes increased with the progression of disease pathology.

### An age-dependent decrease of the VEGF level is accompanied by accumulation of Aβ_1–42_ in the hippocampal blood vessels of OXYS rats

**Fig. 4:**
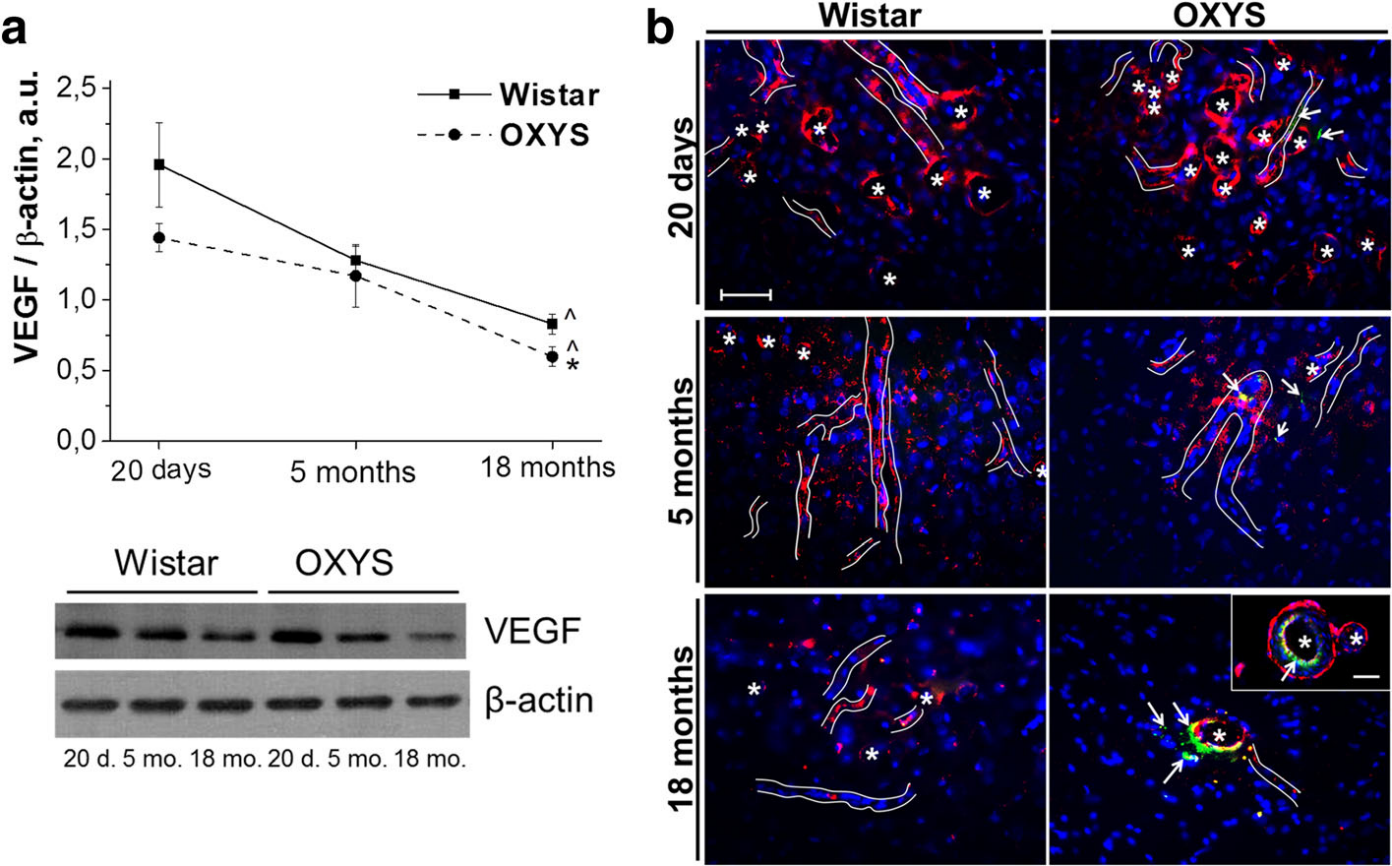
**a** Levels of VEGF decreased with age in the hippocampus of OXYS and Wistar rats with more pronounced decrease in OXYS rats. d. – days; mo. – months. The data are presented as mean ± SEM. ^ - differences with previous age, p < 0.05; * - differences with age-matched Wistar rats, p < 0.05. **b** Immunolabeling for VEGF (red) showed age-related decrease of the number of VEGF-positive vessels in the hippocampus of OXYS and Wistar rats with more pronounced changes in OXYS rats. Immunolabeling for amyloid-β (green) revealed its accumulation with age in the hippocampal vessels of OXYS rats. Borders of VEGF-positive vessels are shown. Asterisks indicate lumens of VEGF-positive capillaries. Arrows indicate amyloid-β content. The DAPI (blue) staining shows cell nuclei. The scale bar is 50 μm

On the basis of the comparative analysis of the functional annotation and pathways, we next examined the concentration of VEGF in the hippocampus of OXYS and Wistar rats at the ages of 20 days and 5 and 18 months. According to western blot analysis (Fig. 4a), the VEGF level in the hippocampus was lower in OXYS rats (F_1,36_ = 5.98, *p* < 0.02). The maximal level of VEGF in the hippocampus of OXYS and Wistar rats was observed at the age of 20 days, and this concentration decreased with age in both rat strains (F_2,36_ = 26.6, *p* < 0.0001), but in OXYS rats, its level at the age of 18 months was lower than that in Wistar rats (*p* < 0.04). Immunolabeling of tissue samples with an anti-VEGF antibody confirmed this result (Fig. 4b). Moreover, immunolabeling for Aβ_1–42_ revealed accumulation of Aβ_1–42_ with age in the hippocampal vessels of OXYS rats and increased colocalization of VEGF with Aβ_1–42_ at the age of 18 months (Fig. 4b).

## Discussion

There is growing evidence of microvascular pathophysiological alterations having a causal role in the development of AD [15]. Our results indicate that cerebrovascular alterations also make a significant contribution to the development of AD-like pathology in OXYS rats. A decline of the hippocampal blood vessel density and ultrastructural abnormalities of these vessels in OXYS rats were revealed in the period of active manifestation of the signs of AD-like pathology, as were significant alterations of these vessels at the progressive stage of the disease, which imply reduced blood flow. Such alteration results in a mismatch between the supply and demand of oxygen and metabolic substrates during the functioning of cerebral tissue; this situation leads to neuronal dysfunction [16]. It should be noted that the signs pointing to a decrease in blood flow were also present in the hippocampus of 5-month-old OXYS rats prior to Aβ deposition, which appears at the age of approximately a year [17] against the background of chronic ischemia and of a decline of cerebrovascular reactivity [11, 12]. A gradual decline in oxygen as well as glucose supply to the brain during aging or hypoxia manifested themselves as contributing factors to hypometabolism. Progression of cerebrovascular alterations impairs vascular clearance of Aβ from the brain and contributes directly to the cognitive symptoms and thereby accelerates the progression of AD [18]. Here we demonstrated that significant cerebrovascular disturbances were observed in the hippocampus of OXYS rats at the age of 18 months, when the cognitive impairments and neurodegenerative processes in the brain were well pronounced and accompanied by significant accumulation of toxic forms of Аβ, formation of amyloid plaques, hyperphosphorylation of the tau protein, mitochondrial dysfunction, and a deficit of synapses [7–9].

Next, we focused on bioinformatic analyses of the data from whole-transcriptome profiling of the hippocampus to identify the molecular mechanism, the metabolic pathways, and biological functions involved in the development of the cerebrovascular dysfunction in OXYS rats. Aging-associated changes occurring at the molecular level contribute to cellular dysfunction and disease. It is important to determine the molecular background of the disease at preclinical stages, which cannot be studied in humans. Our study includes a comparative RNA-Seq analysis of the hippocampal transcriptome between OXYS and Wistar rats at the age of 20 days, which is the period of completion of postnatal development of the brain. By monitoring hippocampal changes of transcript levels at several time points throughout the development of AD-like pathology in OXYS rats, we revealed that already at the age of 20 days, OXYS rats have substantial changes in the expression of 841 protein-coding genes. These early changes before the first clinical signs of AD can serve as the foundation for the development and progression of AD at a later age. It is important that the overwhelming majority of these genes (560) was downregulated in OXYS rats in comparison with age-matched Wistar rats. This trend changed at the age of 5 months when the number of DEGs decreased to 489, and 280 of them were upregulated and were also detected at 18 months of age, when among 1324 DEGs, 750 genes were downregulated, and 574 genes were upregulated in OXYS rats.

In accordance with the purpose of the present study, we analyzed a pool of DEGs that encode gene products that may contribute to the cerebrovascular dysfunction in OXYS rats. In the hippocampus of OXYS and Wistar rats, we identified aging-related DEGs associated with a sufficiently large number of GO terms that are involved in blood vessel processes including blood vessel development, blood circulation, and its regulation. Moreover, the combined analysis of the total list of these DEGs involved in GO terms according to DAVID and in pathways according to KEGG in OXYS and Wistar rats identified similar numbers of DEGs associated with vessel processes. Nonetheless, these processes were represented by different sets of cerebrovascular functions and genes, thereby highlighting a qualitative difference in the age-related alterations of the cerebral vessels between healthy Wistar rats and OXYS rats developing the AD-like pathology. Although between the ages of 20 days and 5 months, genes that exclusively changed expression in OXYS rats were mainly related to blood circulation and regulation of blood pressure, in Wistar rats, such DEGs were related to blood vessel morphogenesis and regulation of angiogenesis. Between the ages of 5 and 18 months, genes exclusively changing expression levels in OXYS rats were mainly related to blood circulation and regulation of blood vessel size, whereas in Wistar rats during the same period, genes that exclusively changed expression were mainly related to the regulation of lymphocyte proliferation and regulation of immune responses. It is noteworthy that 22 of the 32 DEGs associated with the immune system were upregulated. Aging is characterized by chronic low-grade inflammation. Activation of inflammatory reactions could be both beneficial and detrimental to the brain and depends on the magnitude of neurodegenerative changes. In contrast, the imbalance of inflammatory responses in the central nervous system may serve as an initiating factor in many neurodegenerative diseases, including AD [19].

The main aim of this study was to identify the metabolic processes and pathways involved in the development of cerebrovascular dysfunction in OXYS rats. We found that at the preclinical stage of development of the AD-like pathology (age 20 days) as well in the period of its active manifestation (age 5 months) in the hippocampus of OXYS rats, cerebrovascular processes and pathways are upregulated in comparison with Wistar rats; this phenomenon can be considered a compensatory reaction that prevents the development of neurodegenerative processes. These results are consistent with our earlier findings that the neuronal populations in all regions of the hippocampus of OXYS rats increase at the age of 5 months as compared with age-matched Wistar rats owing to the enhanced neurogenesis, which we consider an endogenous compensatory mechanism of repair [7]. During the cerebrovascular dysfunction, elevated neurogenesis associated with increased production of immature neurons failed to integrate the existing networks. Numerous studies have suggested that the dysfunctional neurogenesis in the brain leads subsequently to subtle early manifestations of a disease, which could, in turn, render neurons more vulnerable to AD and contribute to memory impairment [20].

In the hippocampus of OXYS rats, the genes that are differentially expressed during aging were downregulated, and as a result, at the age of 18 months, DEGs were associated with downregulation of cerebrovascular function as compared with Wistar rats. Among these processes, the most significant alterations are associated with blood vessel development, circulatory system processes, and VEGF pathways. VEGF, originally described as a key angiogenic factor, has recently been shown to perform multiple functions in a developing and adult nervous system by acting both on blood vessels and neurons [21]. VEGF has also been shown to serve as a neuroprotective factor in AD. Relative to controls, patients with AD have lower levels of serum VEGF and cerebral capillary VEGF expression in the hippocampus [22].

It is noteworthy that the VEGF signaling pathway in OXYS rats was upregulated at the age of 20 days during completion of postnatal brain development and was downregulated at the age of 18 months: in the period of active progression of the AD-like pathology. The maximal concentration of VEGF in the hippocampus of both OXYS and Wistar rats was detected at the age of 20 days, and this concentration declined with age, becoming lower in OXYS rats than in Wistar rats at the age of 18 months. VEGF has high specificity for endothelial cells and is profoundly accumulated in (and colocalized with) amyloid plaques in the brain of AD patients [23]. We observed increased colocalization of VEGF with Aβ_1–42_ in the hippocampal blood vessels of OXYS rats at age 18 months.

Using network analysis, we identified a core set of 4 interrelated DEGs from functional categories of cerebrovascular dysfunction that were common to 20-day-old and 5- and 18-month-old OXYS rats and probably play a significant role in the development of AD-like pathology in OXYS rats. Three of them (*Nos1*, *P2rx4*, *Pla2g6*) were upregulated at all 3 ages and 1 (*Bdkrb2*) downregulated at the age of 20 days and 18 months, and upregulated at the age of 5 months.

The neuronal nitric oxide synthase NOS1 family, a group of NO synthases that synthesize NO from L-arginine, is a pivotal mediator of neurotransmission, spatial learning, and cognition and has been repeatedly implicated in the neurotoxicity associated with neurodegenerative diseases [24]. A similar NO synthase (endothelial NOS; eNOS), NOS1 plays an obligatory role in the regulation of cerebral blood flow and cell viability and in the protection of nerve cells or fibers from pathogenic factors associated with AD [25]. All three NOS isoforms show aberrant patterns of expression in AD, altering intracellular signaling and routing oxidative stress in directions that are uncompounded [26]; however, data indicating changes in the NOS1 level and their involvement in amplifying or reducing neuronal damage are inconsistent, and to some degree, contradictory. Some studies on different models show overexpression and/or increased activity of nNOS during AD progression [27, 28], whereas others describe reduced nNOS expression and/or activity [29, 30].

The product of the *P2rx4* gene belongs to the family of purinoceptors for ATP. Accumulating evidence reveals major participation of P2 receptors in AD pathology, including Aβ production and elimination, neuroinflammation, neuronal function, and cerebral blood flow [31]. Receptor P2RX4 functions as a ligand-gated ion channel with high calcium permeability and performs critical functions in synaptic plasticity and neuronal survival. Aβ_1–42_ promotes accumulation of calcium-permeable purinergic receptor P2X4 in neurons and thus induces synaptic dysfunction and neuronal death [32]. Experimental evidence from human AD tissue and studies involving selective purinergic receptor agonists and antagonists in vitro and in AD animal models have revealed that purinergic receptors represent novel therapeutic targets in AD [33].

The protein encoded by the *Pla2g6* gene is an A2 phospholipase, a class of enzymes that is known to play multiple roles in the membrane phospholipid homeostasis and in the production of a variety of lipid mediators [34]. *Pla2g6* is the causative gene for a familial form of juvenile-onset dystonia parkinsonism and is associated with the pathogenesis of idiopathic Parkinson’s disease [35]. Reduced phospholipase A_2_ (PLA_2_) activity has been reported in the brain and platelets of patients with AD [36]. In the brain, inhibition of PLA_2_ suppresses the physiological secretion of APP: a mechanism that increases Аβ formation [37].

*Bdkrb2* encodes a receptor for bradykinin (B2R) that elicits many responses including vasodilation, edema, smooth muscle spasm, and pain fiber stimulation [38]. B2R activation may be intimately involved in Aβ-induced neuroinflammation: a key contributor to AD progression. Blockage of B2R can protect against the memory deficits induced by Aβ in mice, pointing to a neuroprotective role of B2R [39]. Here, *Bdkrb2* expression was found to be reduced at preclinical and advanced stages, and was increased at early stage of the AD-like pathology in OXYS rats.

## Conclusions

AD is a multifactorial disease and involves several etiopathogenic mechanisms including cerebrovascular dysfunction. Understanding how neurovascular impairments can lead to AD-related neurodegeneration is necessary to develop effective drugs that prevent or reverse these changes. On the basis of our present and recent results, we can conclude that the development of cerebrovascular impairments in OXYS rats with age along with the age-related decrease in the density of cerebral blood vessels and their structural alterations including accumulation of Aβ, impairment of cerebral blood flow, and increased neuronal degeneration and susceptibility to hypoxia and ischemia. Moreover, we can conclude that changes in the cerebrovascular processes already in the early period of life may contribute in the future to the accelerated cognitive decline in the elderly, and become a factor risk of AD.

## Methods

### Animals and treatments

All the experimental procedures were in compliance with the European Communities Council Directive of 24 November 1986 (86/609/EEC). The protocol of the animal study was approved by the Commission on Bioethics of the Institute of Cytology and Genetics, Novosibirsk, Russia.

To assess the age-dependent transcriptomic changes, male senescence-accelerated OXYS rats (*n* = 12) and age-matched male Wistar rats (n = 12) at the age of 20 days and 5 and 18 months were obtained from the Center for Genetic Resources of Laboratory Animals at the Institute of Cytology and Genetics, the Siberian Branch of the Russian Academy of Sciences (RFMEFI61914X0005 and RFMEFI61914X0010). The OXYS strain was derived from the Wistar strain of rats at the Institute of Cytology and Genetics as described earlier [40] and was registered in the Rat Genome Database (http://rgd.mcw.edu/). At this point, we have the 109th generation of OXYS rats, with spontaneously developing cataract and accelerated senescence syndrome inherited in a linked manner. At the age of 4 weeks, the pups were weaned, housed in groups of 5 animals per cage (57 × 36 × 20 cm), and kept under standard laboratory conditions (22 °C ± 2 °C, 60% relative humidity, and 12-h light/12-h dark cycle; lights on at 9 a.m.). The animals were provided with standard rodent feed (PK-120-1; Laboratorsnab, Ltd., Moscow, Russia) and water ad libitum.

### RNA isolation

For RNA-Seq, 12 male OXYS rats and 12 age-matched male Wistar rats at ages 20 days (*n* = 3), 5 months (n = 3), and 18 months (n = 3) were euthanized by CO_2_ inhalation. After decapitation, the hippocampus was excised rapidly, placed in RNAlater (Ambion, cat. # AM7020), frozen, and stored at − 20 °C prior to analysis. Frozen rat tissues were lysed with the TRIzol Reagent (Invitrogen, Cat. #15596–018), and total RNA was isolated according to the manufacturer’s protocol. RNA quality and quantity were assessed using an Agilent Bioanalyser (Agilent).

### Illumina sequencing

More than 40 million single-end reads 50 bp long were obtained for each sample of hippocampal RNA, using Illumina nonstranded sequencing on an Illumina GAIIx instrument at the Genoanalitika Lab, Moscow [41] in accordance with standard Illumina protocols (mRNA-Seq Sample Prep Kit, cat. # 1004816). Briefly, polyadenylated mRNA was purified from total RNA using Sera-Mag Magnetic Oligo (dT) beads and then broken into small fragments by means of divalent cations and heating. Using a reverse transcriptase and random primers, we synthesized first- and second-strand cDNAs. The cDNA was processed in an end repair reaction with T4 DNA polymerase and Klenow DNA polymerase to blunt the termini. An “A” base was then added to the 3′ end of the blunt phosphorylated DNA fragments, and an Illumina adaptor with a single T overhang at its 3′ end was then ligated to the end of the DNA fragment, for hybridization in a single-read flow cell. After that, a size range of cDNA templates was selected, and these fragments were amplified on a cluster station using the Single-Read Cluster Generation Kit v2. Sequencing-by-synthesis (SBS) at 50-nucleotide length was performed by means of SBS v4 reagents on a Genome Analyzer IIx running the SCS2.8 software (Illumina, cat. #FC-940–4001).

#### Gene expression analysis

The sequencing data were preprocessed using the Cutadapt tool [41] to remove adapters and low-quality sequences. The resulting reads were mapped onto the Rnor_5.0 reference genome assembly in the TopHat2 software [42]. The data were then converted into gene count tables using ENSEMBL and RefSeq gene annotations data. The resulting tables were subjected to the analysis of differential gene expression in the DESeq software [43]. The genes with *p* value < 0.01 were selected as differentially expressed.

### Functional analysis

To identify the GO terms associated with blood vessel processes overrepresented in a DEG list, the detected DEGs were subjected to functional enrichment analyses by means of the DAVID (Database for Annotation, Visualization and Integrated Discovery, https://david-d.ncifcrf.gov/tools.jsp) tool. Pathway analysis of the DEGs associated with blood vessel processes was conducted using the Kyoto Encyclopedia of Genes and Genomes (KEGG) pathways (http://www.genome.jp/kegg/).

### Construction and analysis of gene interaction networks

The gene interaction networks associated with blood vessel processes were identified on the GeneMANIA web server (http://www.genemania.org/) with default parameters. To construct gene interaction networks associated with blood vessel processes from lists of DEGs (raw *p* value < 0.01), we selected genes that were in the following GO categories: blood vessel development, vasculature development, blood vessel morphogenesis, patterning of blood vessels, angiogenesis, blood vessel remodeling, endothelial cell development, vasculogenesis, artery morphogenesis, hemopoiesis, hemopoietic or lymphoid organ development, blood circulation, circulatory system process, vascular process in circulatory system, vasoconstriction, smooth muscle contraction, regulation of blood pressure, positive regulation of smooth muscle cell proliferation, regulation of angiogenesis, regulation of endothelial cell proliferation, regulation of vasoconstriction, positive regulation of endothelial cell proliferation, vascular endothelial growth factor receptor signaling pathway, regulation of smooth muscle cell proliferation according to DAVID and pathways: vascular smooth muscle contraction, VEGF signaling pathway, and hematopoietic cell lineage according to KEGG.

### Western blotting

For this analysis, the hippocampus of 20-day-old (*n* = 6 to 8), 5-month-old (n = 6 to 8), and 18-month-old OXYS and Wistar rats (*n* = 12 to 16) was separated from the brain, placed in a microcentrifuge tube for protein isolation, and frozen in liquid nitrogen. Immunoblotting was carried out as previously described [8]. Antibodies and dilutions used in this study were as follows: anti-VEGF (1:1000; # ab14078, Abcam) and anti-β-actin (1:1000; # ab1801, Abcam) and respective secondary antibodies (1:5000; ## ab6721, ab97135, Abcam). Quantitative densitometric analyses were performed on digitized images of immunoblots in the ImageJ software (NIH, USA).

### Histological examination

The tissue preparation was conducted as described elsewhere [8]. To estimate numeric density of the blood vessels in the middle molecular layer of the dentate gyrus, and in the CA1 and CA3 pyramidal layers of hippocampus, a set of 4–6 serial sections from each animal was used. A 40× objective lens (Axioplan 2, Zeiss) was used to determine the numeric density of capillaries per visual field.

### Immunofluorescent staining

This procedure was performed by a standard indirect method as described previously [8]. Primary antibodies and dilutions were as follows: anti-VEGF (1:250; # ab14078, Abcam) and anti-amyloid-β_1–42_ (1:250; # ab10148, Abcam). After incubation with the respective secondary antibodies conjugated with Alexa Fluor 555 or Alexa Fluor 488 (## ab150170, ab150073, Abcam) diluted 1:250, the tissue slices were coverslipped with the Fluoro-shield mounting medium containing 4′,6-diamidino-2-phenylindole (DAPI; # ab104139, Abcam) and examined under an Axioplan 2 microscope (Zeiss).

### Electron-microscopic examination

For this purpose, the hippocampus samples (*n* = 4) were fixed with 2.5% glutaraldehyde in sodium cacodylate buffer and were prepared as described elsewhere [44]. For evaluation of morphological features, capillaries in the pyramidal layer of the CA1 region were identified in the electron micrographs (30 photos per animal). Next, all the morphological changes that are located in the vessels were painted using software. The photos were processed in Adobe Photoshop. Ultrathin sections were studied and photographed by means of an electron microscope JEM-7A.

### Statistical analysis

The data were subjected to ANOVA (Statistica 8.0 software). Two-way ANOVA was used to evaluate the age-dependent effects (age × genotype [strain]). The Newman–Keuls test was applied to significant main effects and interactions to assess the differences between some sets of means. The data were presented as mean ± SEM. The differences were considered statistically significant at *p* < 0.05.

## Additional files

Additional file 1: The lists of significantly enriched GO terms according to DAVID and pathways according to KEGG results, and the list of significantly differentially expressed protein-coding genes associated with cerebrovascular function identified by the RNA-Seq platform. (XLSX 97 kb)

Additional file 2: The file contains a Venn diagram for significantly differentially expressed protein-coding genes associated with cerebrovascular function. (XLSX 217 kb)

Additional file 3: The file contains significant terms associated with blood vessel processes according to GeneMANIA. (XLSX 47 kb)

## Abbreviations

AD: Alzheimer’s disease
Aβ: Amyloid β
DAVID: Database for annotation, visualization, and integrated discovery
DEG: Differentially expressed gene
GO: Gene ontology
KEGG: Kyoto encyclopedia of genes and genomes
logFC: Log2 fold change
MRI: Magnetic resonance imaging
mRNA: Messenger RNA
RNA-Seq: high-throughput RNA sequencing
VEGF: Vascular endothelial growth factor
